# Establishing a Rapid Enrichment Medium for *Bacillus cereus* to Shorten Detection Time

**DOI:** 10.3390/foods15030466

**Published:** 2026-01-29

**Authors:** Changzheng Shi, Ruirui Hu, Haibo Zhou, Xiaomei Bie, Jun Yang

**Affiliations:** 1 College of Food Science and Technology, Nanjing Agricultural University, Nanjing 210095, China; 2021208002@stu.njau.edu.cn (C.S.);; 2 Key Laboratory of Detection and Traceability Technology of Foodborne Pathogenic Microorganisms, State Administration for Market Regulation, Nanjing 211198, China

**Keywords:** *Bacillus cereus*, rapid enrichment medium, spore germination, detection efficiency

## Abstract

*Bacillus cereus* is a common Gram-positive bacterium that poses a significant threat to food safety due to its environmental adaptability, spore-forming ability, and production of harmful toxins. Traditional detection methods for *B. cereus* are time-consuming and inaccurate. This study aimed to develop a rapid enrichment medium for *B. cereus* to improve detection efficiency. Five *B. cereus* strains and five non-*B. cereus* strains were used. The TSB medium was selected as the basic medium as it supported the best growth and spore germination of *B. cereus* among the tested media. Magnesium sulfate and inosine were identified as the most effective promoters for the growth of vegetative cells and spore germination respectively, while glycine and sodium nitrite were chosen as suitable inhibitors against non-*B. cereus* bacteria. Through orthogonal experiments, the optimal formulation of the rapid enrichment medium (BC-TSB) was determined. BC-TSB effectively inhibited the growth of non-target bacteria and significantly promoted the growth and spore germination of *B. cereus* compared to the TSB basic medium. It also efficiently facilitated the recovery of thermally injured *B. cereus* cells, with a 6 h recovery rate of 87%—shortening the incubation time required by traditional method from 48 h to 6 h. In the detection of artificially contaminated dairy samples, BC-TSB could effectively pre-enrich *B. cereus*, achieving a 100% detection rate in UHT milk, modified milk, and pasteurized milk using both traditional and PMAxx-qPCR methods. Overall, the developed BC-TSB medium has great potential for the rapid and accurate detection of *B. cereus* in food, which can help enhance food safety monitoring.

## 1. Introduction

*Bacillus cereus* is a common Gram-positive pathogenic bacterium that can produce spores and various harmful toxins, posing an undeniable threat to human health [1]. The *B. cereus* exhibits environmental adaptability and resistance to factors such as temperature, pH, desiccation, and irradiation owing to its capacity to form thick-walled spores and express stress-responsive genes, enabling it to survive on various environmental surfaces and materials [2]. Notably, its primary natural reservoirs include soil, plant surfaces, and aquatic environments, where it persists as dormant spores and serves as the primary source of subsequent contamination. Food is one of the important vehicles of *B. cereus* contamination, especially grains, pasta, dairy products, and pastries [3]. *B. cereus* can produce enzymes like proteases, lipases, and lecithinases, which can break down the major components in milk, such as proteins, lipids, and lecithin [4]. Based on toxin-producing characteristics, *B. cereus* produces two main categories of toxins associated with foodborne diseases, namely the emetic toxin cereulide and a suite of enterotoxins (e.g., hemolysin BL [Hbl], nonhemolytic enterotoxin [Nhe], and cytotoxin K [CytK]). The bacterium can thus be classified into an emetic type due to the production of the emetic toxin cereulide and a diarrheal type caused by the production of enterotoxins [1]. The emetic toxin cereulide is only found in a specific subgroup of *B. cereus*, while enterotoxin genes and other virulence factors are widely distributed among all members of *B. cereus* [5]. Since *B. cereus* is widespread in food and the emetic toxin is highly resistant to high temperature and acidic environment [6], it poses a significant threat to food safety.

*B. cereus* can form biofilms and spores, which are important causes of *B. cereus*-related food poisoning outbreaks. Biofilms serve as a reservoir for spores, there is a synergistic effect among spore formation, toxin production, and biofilm formation, thus creating serious safety risks [7]. During food processing, treatment methods can cause microorganisms to assume different physiological states: undamaged cells, sublethally damaged cells, and lethally damaged cells [8]. Sublethally damaged cells can recover from their injuries and resume growth under suitable conditions (selective media), which may lead to false-negative results in traditional culture-based detection methods [9]. According to the standard ISO 7932:2004 [10], Mannitol-Yolk Polymyxin (MYP) is used as a selective agar for counting *B. cereus* [11]. However, when testing samples with a large variety and quantity of bacteria using MYP, the diffusion and overlap of lecithin precipitation rings and the cross-linking of colonies can cause some non-target bacteria to ferment mannitol, “turning” the entire MYP plate yellow, making it impossible to accurately identify and count the bacteria [12].

The main traditional method for detecting *B. cereus* is the bacterial isolation, culture, and identification method. This process is rather cumbersome and usually requires a long-term enrichment culture and selective isolation [13]. Given that rapid and accurate detection of *B. cereus* is critical for real-time food safety supervision and timely response to foodborne disease outbreaks caused by this pathogen, in this study, a highly efficient and rapid enrichment medium for *B. cereus* was developed. By screening appropriate promoters and inhibitors through orthogonal experiments, this medium can promote spore germination, rapidly recover from sublethal states, and more effectively inhibit the growth of miscellaneous bacteria other than *B. cereus* compared with conventional media. It can significantly reduce the time required for the enrichment and detection of *B. cereus* and improve the accuracy of detection, thereby providing robust technical support for safeguarding dairy product safety and controlling potential public health risks.

## 2. Materials and Methods

### 2.1. Strains in This Study

Five *B. cereus* (*B. cereus* CCTCC AB 2010134, *B. cereus* CCTCC AB 204038, *B. cereus* CMCC 63301, *B. cereus* IS3 (isolated strains from our laboratory), *B. cereus* IS9 (isolated strains from our laboratory)) served as positive strains, while five non-*B. cereus* strains (*Salmonella enterica* serotype Typhimurium CICC 21483, *Escherichia coli* O157:H7 CICC 21530, *Listeria monocytogenes* CICC 21662, *Vibrio parahaemolyticus* CICC 21617, *Staphylococcus aureus* CMCC 26074) were used as negative strains. These non-*B. cereus* strains were typical foodborne pathogens that frequently co-contaminate foods with *B. cereus* in practical scenarios, covering both Gram-positive and Gram-negative groups to ensure the comprehensiveness of specificity verification, and were standard strains with well-characterized phenotypes. All strains were stored at −80 °C in our laboratory in Tryptic Soy Broth (TSB) supplemented with 20% (*v*/*v*) glycerol as the cryoprotectant. Prior to the experiment, they were cultured aerobically in TSB medium at 37 °C with 180 rpm for 12 h for activation. MYP agar medium, polymyxin B, LB medium (Luria–Bertani medium), BHI medium (Brain Heart Infusion medium), NB medium (Nutrient Broth medium), and TSB medium were purchased from Hope Bio-Technology Co., Ltd. (Qingdao, China).

### 2.2. Screening of Basal Media for Promoting the Growth of B. cereus

The cultivation and counting of *B. cereus* were performed according to a previous method with minor modifications [14]. A single colony of *B. cereus* was picked into TSB medium and incubate it overnight at 37 °C. The OD_600_ of the bacterial suspension was adjusted to 0.4 to obtain the seed culture. Subsequently, l mL seed culture was inoculated into 100 mL of each liquid medium (LB, BHI, NB, TSB, and 2% NB). The inoculated media were incubated at 37 °C for 12 h. 1 mL of the cultured bacterial solution was taken, and ten-fold serial dilutions were performed with 0.85% sterile physiological saline. An appropriate dilution was selected for inoculation onto solid plates (LB, BHI, NB, TSB, and 2% NB), and the plates were incubated aerobically at 37 °C for 12 h for colony counting. The number of colonies was maintained within the range of 30–300.

### 2.3. Growth Curve Plotting and Post-Germination Recovery of B. cereus Spores’ Assay

The growth curve of *B. cereus* was plotted to determine the spore-forming period, which laid a foundation for the subsequent screening of media that promote spore germination. The *B. cereus* seed solution was inoculated into the optimal basal medium at a ratio of 1%, incubate at 37 °C, and the OD_600_ value was measured every 2 h.

The spore suspension of *B. cereus* was prepared according to a previously published method with minor modifications [15]. The five *B. cereus* strains were activated by picking a single colony and inoculating it into liquid medium for 12 h incubation. After culturing in TSB medium at 37 °C for 30 h, a duration validated to induce *B. cereus* entry into the late stationary phase with abundant spore formation [16], 10 mL aliquots of the culture were subjected to an 80 °C digital constant-temperature water bath (model: HH-1, volume: 1.5 L) for 20 min to kill the vegetative cells and retain the spores. Spores were harvested by centrifugation at 3345× *g* for 20 min at 4 °C and washed 3 times with sterile distilled water. The spore pellet was then resuspended in 2 mL of sterile distilled water. Subsequently, the spore solution was inoculated into LB, BHI, NB, TSB, and 2% NB media at an inoculation rate of 2%, and incubated at 37 °C for 18 h for colony counting.

### 2.4. Optimization of the Addition Amounts of Promoters and Inhibitors

Promoters and inhibitors selection was performed according to previously described method with minor modifications [17]. In this study, magnesium sulfate, potassium dihydrogen phosphate, corn starch, and sodium dihydrogen phosphate were selected as promoters for the growth of bacterial cells, while ammonium sulfate, manganese sulfate, L-alanine, and inosine were chosen as promoters for spore germination. Bile salts, glycine, ethylenediaminetetraacetic acid (EDTA), sodium deoxycholate, nalidixic acid, sodium nitrite, magnesium chloride, ε-polylysine, and cinnamaldehyde were selected as inhibitors. All the aforementioned reagents were purchased from Sinopharm Chemical Reagent Co., Ltd. (Shanghai, China). The growth promoters, inhibitors, and spore germination promoters were added to 100 mL TSB medium according to the amounts shown in Table S1, respectively. Then, the target bacteria, *B. cereus*, non-*B. cereus* bacteria, and spores of *B. cereus* were inoculated separately. After culturing at 37 °C for 10 h, the OD_600_ value was measured. Promotion rate and inhibition rate was calculated according to Formula (1) and Formula (2) respectively.

(1) $$\mathrm{Promotion}\;\mathrm{rate}\;(\%)=\left(\frac{{\mathrm{OD}}_{600}\left(\mathrm{TSB}\;\mathrm{with}\;\mathrm{promoter}\right)}{{\mathrm{OD}}_{600}\left(\mathrm{TSB}\;\mathrm{without}\;\mathrm{promoter}\right)}\;-\;1\right)\;\times \;100\%$$

(2) $$\mathrm{Inhibition}\;\mathrm{rate}\;(\%)=\left(1-\frac{{OD}_{600}(TSB\;with\;inhibitor)}{{OD}_{600}(TSB\;without\;inhibitor)}\right)\;\times \;100\%$$

Two promoters with the best effects on *B. cereus* growth and spore germination, and two inhibitors that exhibited the best inhibitory effects on non-*B. cereus* bacteria while having relatively little impact on *B. cereus*, were selected from the above-mentioned substances. An orthogonal experiment with four factors and four levels was conducted, and the optimal combination was selected via the range analysis method.

### 2.5. Establishment of the Thermal Injury Model of B. cereus and Determination of the Recovery Effect

The thermal injury model of *B. cereus* was established according to previous method with minor modification [8]. In order to construct a thermal injury model of *B. cereus*, the *B. cereus* bacterial suspension (approximately 1 × 10^8^ CFU) was subjected to heat treatment at 72 °C for 10 s to 50 s, and at 75 °C for 15 s to 55 s. After diluting with sterile physiological saline, 100 μL of the appropriately diluted suspension was taken and inoculated onto the selective enrichment solid medium (MYP agar plate) and TSB solid medium (TSA) using the spread plating method. Plates were incubated at 37 °C for 24 to 48 h, and colonies were then counted. The mortality rate and sub-lethal rate of *B. cereus* were calculated according to Formulas (3) and (4).

(3) *Mortality rate* = (1 − *T*_0_/*T*_1_) × 100%

(4) *Sub-lethal rate* = (1 − *M*_0_/*M*_1_) × 100%

Note: *T*_0_ represents the number of colonies of *B. cereus* on the TSA plate after thermal injury, and *T*_1_ represents the number of colonies of *B. cereus* on the TSA plate without thermal injury. *M*_0_ represents the number of colonies of *B. cereus* on the MYP plate after thermal injury, and *M*_1_ represents the number of colonies of *B. cereus* on the MYP plate without thermal injury. The unit of the number of colonies is CFU/mL.

Thermally injured *B. cereus* was separately inoculated into TSB, BC-TSB (the optimized TSB medium) and TSPB (TSB containing 100 IU/mL polymyxin B), and cultured at 37 °C for 6 h. After dilution, the bacterial suspension was spread onto TSA plates and MYP plates, which were then incubated at 37 °C for 24 h to 48 h for colony counting. The recovery states of thermally injured *B. cereus* in BC-TSB, TSB and TSPB were compared.

### 2.6. Detection of Artificially Contaminated Dairy Samples by B. cereus

To evaluate the enrichment ability of BC-TSB for *B. cereus* in milk with a relatively high number of competing microbiota, commercially available UHT sterilized milk, pasteurized milk, formulated milk, and fermented milk were purchased as samples. The milk products were confirmed free of pathogens using standard culture methods (Food and Drug Administration (FDA) Bacteriological Analytical Manual (BAM), Chapter 14) [18]. *B. cereus* was inoculated into the samples with a final concentration of 10^1^ CFU/mL. 25 mL of the inoculated sample was added to 225 mL of BC-TSB and TSPB media respectively, and cultured at 37 °C for 6 h. Both the traditional culturing method and the PMAxx-qPCR method developed in our laboratory were used for detection [19]. Briefly, 500 μL of bacterial suspension was mixed with 40 μmol/L PMAxx (Biotium Inc., Fremont, California (CA), USA) and incubated in the dark at 37 °C for 5 min. Subsequently, the sample was exposed to a 500 W halogen lamp (20 cm distance) for 9 min to inactivate excess PMAxx. Total DNA was extracted via thermal lysis (100 °C for 20 min, followed by centrifugation at 12,000× *g* for 5 min to collect supernatant) and subjected to qPCR using the kit’s reaction master mix.

### 2.7. Statistical Analysis

All of the above experiments were conducted in triplicate, and all values are expressed as the means ± standard deviations (SD). Data were analyzed using ANOVA, followed by Tukey test to determine the significant difference between the means using SPSS 20.0 software. *p* < 0.05 was considered statistically significant. All figures in this study were generated using GraphPad Prism 8 (GraphPad Software, LLC, Boston, MA, USA) and BioRender (available online at https://www.biorender.com).

## 3. Results

### 3.1. Nutrient Media Screening for B. cereus

**Figure 1:**
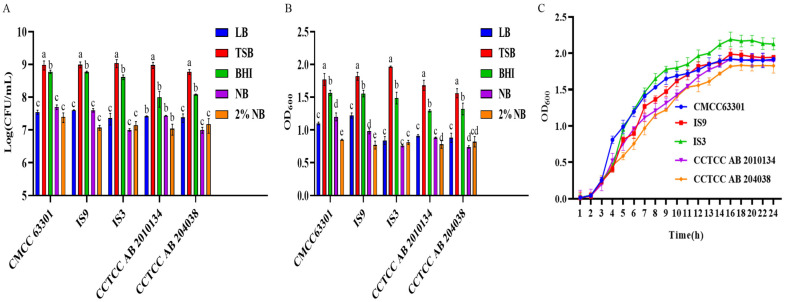
Determination of the growth abilities of five *B. cereus* strains in different media. (**A**) The growth abilities of five *B. cereus* strains in different media were determined by the plate-counting method. (**B**) The growth abilities of five *B. cereus* strains in different media were evaluated by measuring the absorbance. (**C**) The growth curves of five *B. cereus* strains in TSB medium. Different lowercase letters indicate significant differences within the group (*p* < 0.05).

Five kinds of nutrient media were used to culture *B. cereus*. Plate counting (Figure 1A) and determination of the OD_600_ value (Figure 1B) were carried out respectively to evaluate the influence on the growth ability of *B. cereus*. The results showed that different strains of *B. cereus* could all grow in the five kinds of media, but there were differences in their growth abilities. *B. cereus* possessed the strongest growth ability in the TSB medium, and there was a significant difference compared with other media (*p* < 0.05). Therefore, the TSB medium was selected as the basic medium in this study. The growth curves of 5 strains of *B. cereus* were determined, all of which showed similar changing trends (Figure 1C). The 5 strains of *B. cereus* entered the logarithmic growth phase after 2 h, reached the maximum OD_600_ value after 16 h, and entered the stationary phase. During the logarithmic growth phase, the reproduction of *B. cereus* was greater than its death, and its physiological characteristics were stable. This stage was selected as the inoculation period for the subsequent single-factor experiments. Spores generally formed in large numbers in the later stage of the stationary phase, and the stage of 30 h of culture was selected as the stage for spore collection.

### 3.2. Effect of Basal Media on Post-Germination Recovery of Bacillus cereus Spores

**Figure 2:**
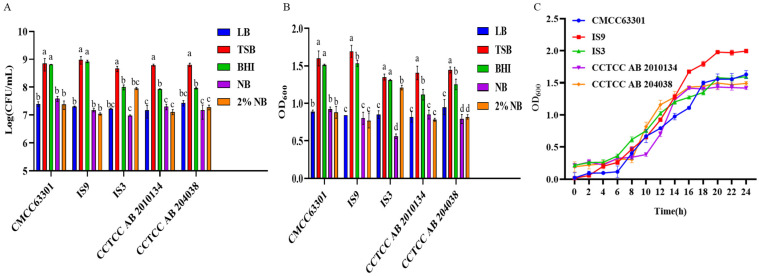
Determination of the germination abilities of spores from five *B. cereus* strains in different media. (**A**) The spore germination abilities of five *B. cereus* strains in different media were determined by the plate-counting method. (**B**) The spore germination abilities of five *B. cereus* strains in different media were evaluated by measuring the absorbance. (**C**) The growth curves of spores from five *B. cereus* strains in TSB medium. Different lowercase letters indicate significant differences within the group (*p* < 0.05).

The bacterial suspension of *B. cereus* cultured to the later stage of the stationary phase was placed in a water bath at 80 °C for 20 min to destroy the vegetative cells and retain the spores. The spore suspension was inoculated into 5 kinds of basic media. Through plate counting (Figure 2A) and determination of the OD_600_ value (Figure 2B), the effect of the basic media on the spore germination of *B. cereus* was evaluated. The results showed that the TSB and BHI media had better effects on the *B. cereus* spore germination. Moreover, the spore germination effects of *B. cereus* CCTCC AB 2010134 and CCTCC AB 204030 in the TSB medium were significantly better than those in the BHI medium (*p* < 0.05). Therefore, the TSB medium was selected as the basic medium for the spore germination of *B. cereus*. Then, the germination and growth curve of the spores in the TSB medium was determined (Figure 2C). The results showed that the strain remained in the lag phase from inoculation to 6 h of culture, entered the logarithmic growth phase at 6 h post-inoculation, and its growth slowed after approximately 18 h, with the strain entering the stationary phase. Compared with the vegetative cells, the growth rate of the spores was slow, but after the spores germinated, they could grow and reproduce rapidly and enter the stationary phase.

### 3.3. Selection of Promoters for Target Bacteria and Inhibitors for Non-Target Bacteria

**Figure 3:**
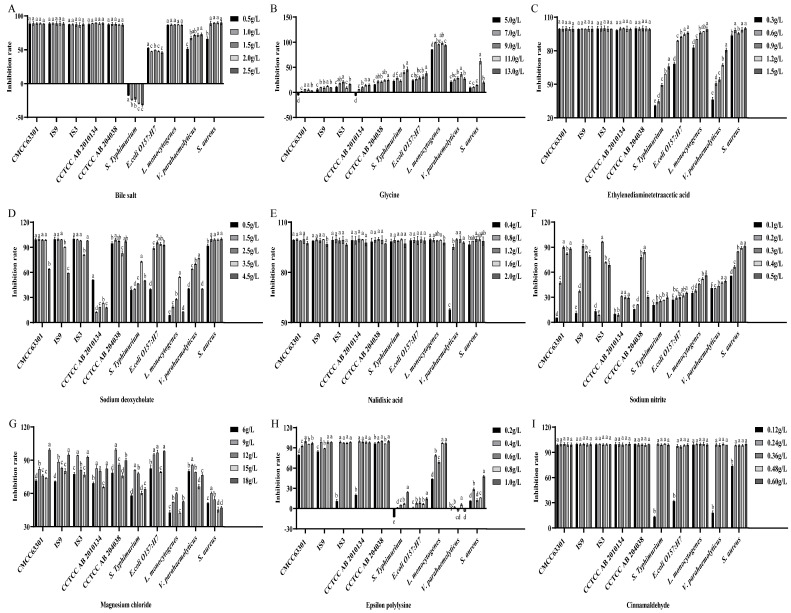
Effects of different inhibitors on the growth of *B. cereus* and non-*B. cereus* bacteria. (**A**) Bile salts. (**B**) Glycine. (**C**) EDTA. (**D**) Sodium deoxycholate. (**E**) Nalidixic acid. (**F**) Sodium nitrite. (**G**) Magnesium chloride. (**H**) Epsilon polylysine. (**I**) Cinnamaldehyde. Different lowercase letters indicate significant differences within the group (*p* < 0.05).

In this study, nine substances were selected as candidate inhibitors, which were required to inhibit the growth of other non-target bacteria except *B. cereus* (significant inhibition: inhibition rate ≥ 50%), but had little impact on the target bacteria (low inhibition: inhibition rate < 20%). The results showed that bile salts (Figure 3A), EDTA (Figure 3C), sodium deoxycholate (Figure 3D), nalidixic acid (Figure 3E), magnesium chloride (Figure 3G), ε-polylysine (Figure 3H) and cinnamaldehyde (Figure 3I) had a high inhibition rate against *B. cereus*, so they were not suitable as inhibitors. Sodium nitrite at a concentration of 0.1 g/L had a low inhibition rate against *B. cereus*, and had a significant inhibitory effect on *S. enterica* serotype Typhimurium, *E. coli* O157:H7, *L. monocytogenes* and *V. parahaemolyticus*. Among them, the inhibition rate against *S. aureus* reached 55.65% (Figure 3F), and it could be used as an effective candidate inhibitor. Glycine also showed a good inhibitory effect. Glycine at a concentration of 11 g/L had a low inhibition rate against *B. cereus*, but had a good inhibitory effect on non-target bacteria. The inhibition rates against *S. enterica* serotype Typhimurium, *E. coli* O157:H7 and *V. parahaemolyticus* were all around 40%, the inhibition rate against *S. aureus* exceeded 60%, and the inhibition rate against *L. monocytogenes* was the highest, reaching 98.3% (Figure 3B).

**Figure 4:**
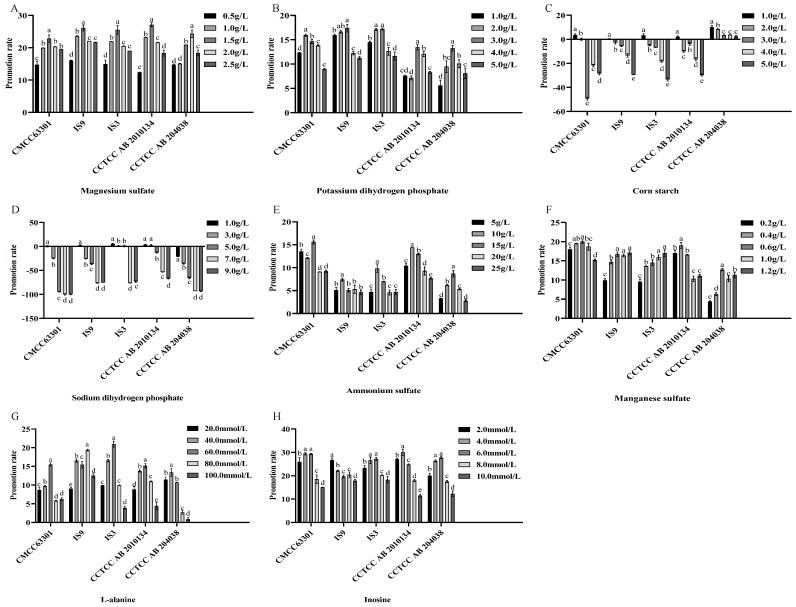
Effects of different promoters on the growth ability of *B. cereus* vegetative cells and the spore germination ability. (**A**–**D**) show the effects of promoters on the growth ability of *B. cereus* vegetative cells. (**A**) Magnesium sulfate. (**B**) Potassium dihydrogen phosphate. (**C**) Corn starch. (**D**) Sodium dihydrogen phosphate. (**E**–**H**) show the effects of promoters on the spore germination and growth of *B. cereus*. (**E**) Ammonium sulfate. (**F**) Manganese sulfate. (**G**) L-alanine. (**H**) Inosine. Different lowercase letters indicate significant differences within the group (*p* < 0.05).

The selection of promoters included the promotion of the growth of *B. cereus* vegetative cells (Figure 4A–D) and the promotion of spore germination (Figure 4E–H). Magnesium sulfate at a concentration of 1.5 g/L had the most obvious growth-promoting effect on *B. cereus* (Figure 4A), and the promotion rates for the five *B. cereus* strains all reached more than 20%. Potassium dihydrogen phosphate also showed a similar effect, but the promotion rate only reached about 15% (Figure 4B). However, when the concentrations of corn starch (Figure 4C) and sodium dihydrogen phosphate (Figure 4D) increased, the promotion rates decreased, and the growth of *B. cereus* was inhibited. Ammonium sulfate (Figure 4E), manganese sulfate (Figure 4F), L-alanine (Figure 4G) and inosine (Figure 4H) all had a significant promoting effect on spore germination. Among them, inosine had a high promotion rate for spore germination. The promotion rates of inosine at a concentration of 2–8 mmol/L for the spore germination of different strains of *B. cereus* reached about 30%, and it was the most suitable spore germination promoter.

### 3.4. Optimization of the Formulation of Rapid Enrichment Medium

In order to optimize the formulation of the rapid enrichment medium, an orthogonal experiment with 4 factors and 4 levels was designed. The growth of vegetative cells and the promotion of spore germination were used as the indicators respectively (Table S2). The results showed that factor B (Magnesium sulfate) led to the largest range of the results, indicating that magnesium sulfate is the main factor affecting the growth of vegetative cells and spore germination of *B. cereus*. According to the analysis of the results of the orthogonal experiment, 6 mmol/L inosine, 2 g/L magnesium sulfate, 5 g/L glycine and 0.3 g/L sodium nitrite had the best effect on the growth of vegetative cells of *B. cereus*. For the spore germination of *B. cereus*, 8 mmol/L inosine, 1.5 g/L magnesium sulfate, 7 g/L glycine and 0.1 g/L sodium nitrite had the best effect. Through comprehensive integration and analysis of the above two sets of results, the final optimal formulation of the enrichment medium was established as 8 mmol/L inosine, 2 g/L magnesium sulfate, 7 g/L glycine, and 0.3 g/L sodium nitrite. The optimized TSB-based enrichment medium with this formulation was designated as BC-TSB.

### 3.5. Promoting effects of BC-TSB on bacterial growth and spore germination

**Figure 5:**
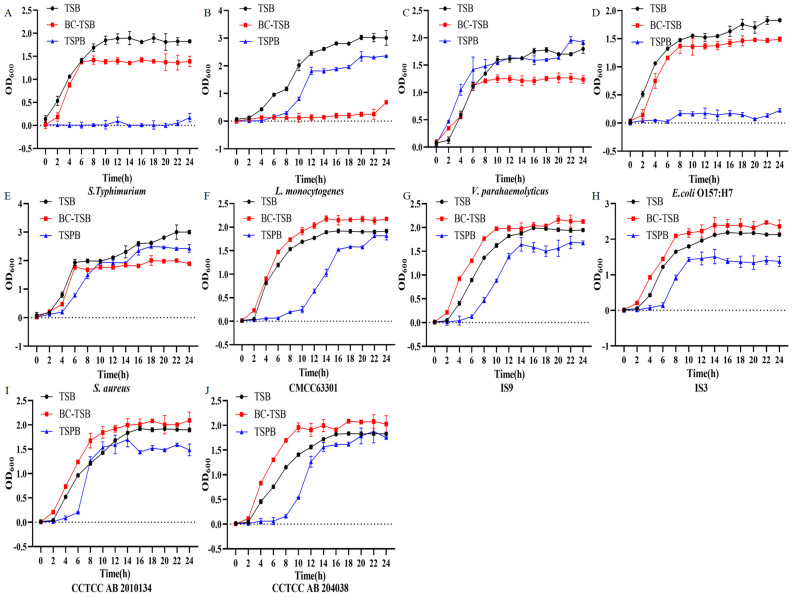
Determination of the growth abilities of *B. cereus* and non-*B. cereus* bacteria in different media. BC-TSB represents the optimized TSB medium, and TSPB represents the TSB medium containing polymyxin B. (**A**) *S. enterica* serotype Typhimurium. (**B**) *L. monocytogenes*. (**C**) *V. parahaemolyticus*. (**D**) *E. coli* O157:H7. (**E**) *S. aureus*. (**F**) *B. cereus* CMCC63301. (**G**) *B. cereus* IS9. (**H**) *B. cereus* IS3. (**I**) *B. cereus* CCTCC AB 2010134. (**J**) *B. cereus* CCTCC AB 204038.

**Figure 6:**
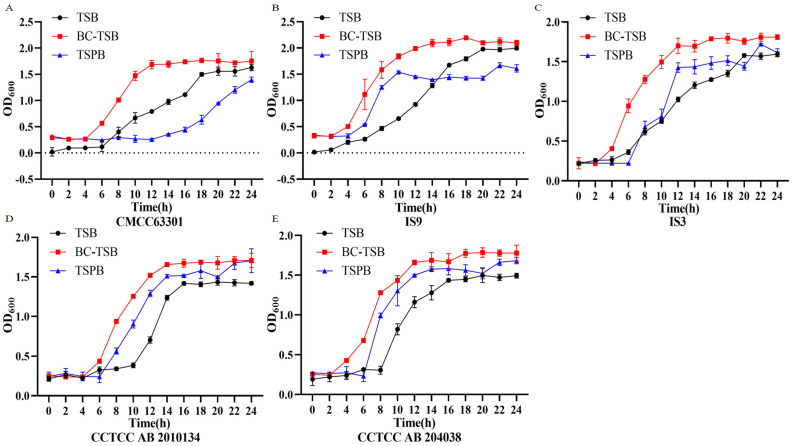
Determination of the germination ability of *B. cereus* spores in different media. (**A**) *B. cereus* CMCC63301. (**B**) *B. cereus* IS9. (**C**) *B. cereus* IS3. (**D**) *B. cereus* CCTCC AB 2010134. (**E**) *B. cereus* CCTCC AB 204038.

The effects of TSB basal medium, optimized TSB medium (BC-TSB), and TSB medium supplemented with polymyxin B (TSPB) on *B. cereus* growth and spore germination were analyzed, alongside their differential impacts on the growth of non-target bacteria. The results showed that TSPB could significantly inhibit the growth of *S. enterica* serotype Typhimurium and *E. coli* O157:H7 (Figure 5A,D). BC-TSB could effectively inhibit the growth of non-target bacteria, and the OD_600_ value in the stationary phase was significantly lower than that of the TSB basic medium. *L. monocytogenes* hardly grew in the BC-TSB medium (Figure 5B). It is worth noting that the BC-TSB medium could promote the growth of 5 strains of *B. cereus*, while the TSPB medium led to a decrease in the growth ability of *B. cereus*. The results indicate that BC-TSB has a relatively obvious promoting effect on the growth of the target bacteria, achieving the goal of rapid enrichment of bacteria. The results in Figure 6 show that the BC-TSB medium can rapidly promote the spore germination of *B. cereus*, shortening the germination time, and the effect is significantly better than that of the TSB basic medium.

### 3.6. Thermal Injury Recovery of B. cereus and Detection in Artificially Contaminated Samples

**Figure 7:**
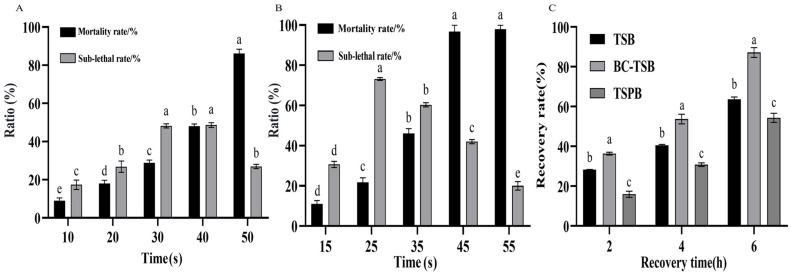
Establishment and recovery of thermal injury in *B. cereus*. (**A**) Effects of 72 °C treatment on the mortality and sublethal rates of *B. cereus*. (**B**) Effects of 75 °C treatment on the mortality and sublethal rates of *B. cereus*. (**C**) Recovery of thermal injury *B. cereus*. Different lowercase letters indicate significant differences within the group (*p* < 0.05).

First, a thermal injury model of *B. cereus* was established. The *B. cereus* culture was treated in a water bath at 72 °C (Figure 7A) and 75 °C (Figure 7B). The results showed that the mortality rate caused by heat treatment increased as the treatment time extended. Conversely, the sublethal rate initially increased and then decreased with the prolongation of the treatment time. In order to establish a thermal injury model, the treatment conditions with a relatively high sublethal rate but a relatively low mortality rate should be selected. Therefore, a thermal injury model was constructed by heat treatment at 75 °C for 25 s. The recovery effects of TSB medium, BC-TSB medium, and TSPB medium on thermally injured *B. cereus* were determined. The results showed that with the extension of the recovery time, the recovery rate of thermally injured cells increased. The recovery rate of thermally injured cells in the BC-TSB medium reached 87% at 6 h, which had significant difference from the recovery rates in the TSB and TSPB media (Figure 7C). The results indicate that the BC-TSB medium can efficiently recover the thermally injured cells of *B. cereus*.

**Figure 8:**
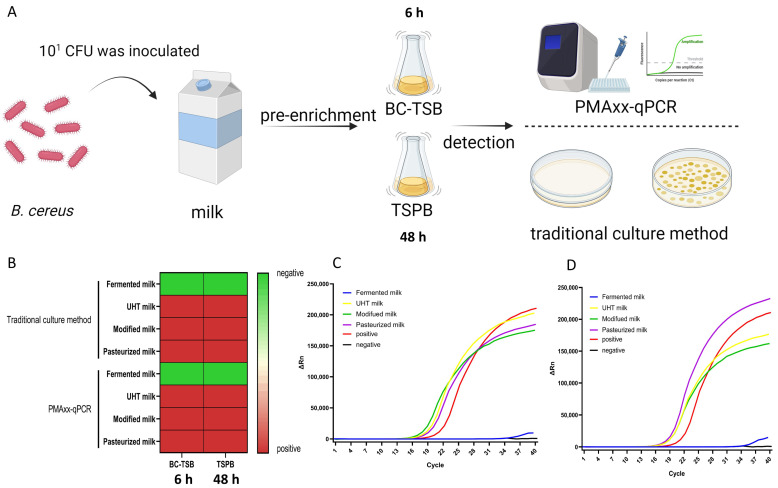
Artificial contamination experiment for the detection of *B. cereus* in milk. (**A**) Artificial contamination experiment process. *B. cereus* was inoculated into milk, followed by enrichment culture (BC-TSB for 6 h and TSPB for 48 h), and then detected using the PMAXX-qPCR and traditional culture methods. (**B**) Detection results of *B. cereus* in milk. Red indicates *B. cereus* is detected (pos-itive), and green indicates it is not detected (negative). (**C**) Amplification curves of PMAxx-qPCR for *B. cereus* detection in different milk samples after 6 h enrichment with BC-TSB. (**D**) Amplification curves of PMAxx-qPCR for *B. cereus* detection in different milk samples after 48 h enrichment with TSPB. ΔRn denotes the relative change in fluorescent signal in the qPCR reaction, and the abscissa represents qPCR reaction cycle number.

10^1^ CFU of *B. cereus* was inoculated into dairy products to simulate artificially contaminated samples. BC-TSB and TSPB media were used for pre-enrichment culture respectively, and *B. cereus* was detected by traditional culture methods and the PMAxx-qPCR method developed in our laboratory (Figure 8A). The results showed that viable *B. cereus* was not detected in fermented milk. It was suggested that the acidic environment of fermented milk was not suitable for the survival of *B. cereus*, resulting in the death of artificially contaminated *B. cereus*. After enrichment in BC-TSB and TSPB media, the detection rates of *B. cereus* in UHT milk, modified milk and pasteurized milk were 100% by both traditional detection methods and the PMAxx-qPCR method (Figure 8B). The PMAxx-qPCR amplification curves after BC-TSB enrichment and TSPB enrichment showed obvious fluorescent signal amplification of *B. cereus* in these three milk samples (Figure 8C,D). The results indicate that BC-TSB can effectively perform pre-enrichment treatment on *B. cereus*, facilitating subsequent detection.

## 4. Discussion

*B. cereus* is widely survived in the environment and is an opportunistic pathogen. Due to the presence of spores, once it is introduced into the food production process, it is difficult to eliminate [20]. Therefore, it is of great significance to establish a rapid and accurate detection method. The pre-enrichment step before detection is a critical component of the detection workflow: food samples often contain *B. cereus* at concentrations below the limit of detection of direct assays, and sublethally damaged *B. cereus* cells cannot form colonies or be detected directly. Pre-enrichment thus provides the necessary conditions for their recovery and proliferation. Shortening the time of the pre-enrichment step can greatly improve the detection efficiency. Traditional pre-enrichment media often lack targeted promotion of *B. cereus* spore germination and suffer from slow enrichment rates [21]. In this study, an optimized TSB medium was developed, which has the dual functions of promoting spore germination and bacterial enrichment.

Among the eight promoters used in this study, magnesium sulfate had the most obvious effect on the enrichment of *B. cereus*. Magnesium ions are important cofactors for many enzymes and essential nutrients required for bacterial growth and cell maintenance, influencing cell attachment and aggregation [22]. The addition of magnesium ions can be used to regulate the acetylation of proteins during microbial growth, potentially reducing the potential harmful acetylated isomers in recombinant proteins without having a negative impact on cell growth [23]. Makkar et al. demonstrated that magnesium sulfate enhances *B. cereus* growth [24]. Studies have found that D-glucose, L-alanine, and inosine can independently induce *B. cereus* endospore germination [25]. Additionally, inosine at 0.01 to 10 mmol/L or L-alanine at 1 to 100 mmol/L has been shown to effectively promote the germination of *B. cereus* spores [25]. Inosine promotes *B. cereus* spore germination by binding to the gerR-encoded receptor, triggering the germination cascade [26]. Our results showed that 2–8 mmol/L of inosine could enable the spores to germinate rapidly in a short period. Glycine can inhibit the synthesis of the cell wall, thus exhibiting antibacterial activity [27], and 0.5% glycine has been reported to inhibit the growth of *Bacillus subtilis* through the same cell wall synthesis interference mechanism [28]. Nitrite has a good inhibitory effect on various microorganisms such as *L. monocytogenes*, *S. aureus*, and *Salmonella* [29], and its combined use with sodium chloride can effectively inhibit the spore germination of *Clostridium beijerinckii* [30]. This study demonstrated that 11 g/L of glycine and 0.5 g/L of sodium nitrite have a good inhibitory effect on non-target bacteria without affecting the growth of the target bacteria.

Microbial cells with sublethal damage can regain their activity under certain conditions. However, failure to grow and reproduce under inappropriate culture conditions may lead to their misclassification as non-viable [31]. Damaged sublethal cells are unable to form colonies on selective media and require specific culture conditions to restore their physiological state for the detection of sublethal cells [32]. Previous studies have found that BHI and rice broth support the recovery of heat-damaged cells of *B. cereus*, with the recovery rate reaching nearly 100% following 3 days of culture [33]. In contrast, the BC-TSB selective enrichment medium developed in this study can meet the nutritional requirements of *B. cereus* during the recovery process. It achieves 87.20% recovery of the target bacteria within 6 h, and the recovery effect is significantly better than that of TSPB and TSB. After the *Salmonella* enrichment medium recovered *S. enterica* for 5 h, the damage rate decreased from 62.02% to 7.54%.

Dairy products are nutritionally rich but highly susceptible to pathogenic bacterial contamination and deterioration. Among them, *B. cereus* has a relatively high detection rate in raw milk; its spores can germinate and proliferate extensively in dairy products, leading to decreased pH values, excessive microbial counts, and thus severe impacts on product shelf life [2]. In this study, artificial contamination experiments were conducted on four common dairy products (fermented milk, UHT milk, modified milk, and pasteurized milk). Notably, the final inoculation concentration of *B. cereus* was set to 10^1^ CFU/mL, which is consistent with the actual low contamination level of *B. cereus* in real dairy product processing and storage environments (typically 10^0^–10^2^ CFU/mL). This concentration setting avoids the artificial high bacterial load that deviates from practical scenarios, ensuring the ecological relevance and practical application value of the experimental results for food safety testing. *B. cereus* was successfully detected in UHT milk, modified milk, and pasteurized milk. Notably, no *B. cereus* was detected in fermented milk, which can be attributed to the unique characteristics of fermented milk production and composition. The absence of *B. cereus* in fermented milk is mainly due to the following factors: first, fermented milk undergoes fermentation by probiotics such as lactic acid bacteria, which produce large amounts of organic acids during metabolism, significantly reducing the product’s pH value. This acidic environment not only inhibits the germination of *B. cereus* spores but also suppresses the growth and reproduction of vegetative cells [34]. Second, probiotics in fermented milk can secrete antibacterial substances that exert direct inhibitory or bactericidal effects on *B. cereus* [35]. Additionally, the intense nutritional competition between probiotics and *B. cereus* further limits the survival of *B. cereus* in fermented milk. To address the challenge of *B. cereus* detection in contaminated dairy products, we optimized the enrichment medium (BC-TSB). This optimized medium can effectively accelerate the germination of *B. cereus* spores in UHT milk, modified milk, and pasteurized milk. Importantly, the BC-TSB medium enables accurate detection of *B. cereus* in just 6 h, which greatly improves detection efficiency and shortens the overall detection process, providing a rapid and reliable technical support for the quality control of dairy products.

## 5. Conclusions

In this study, we successfully developed an optimized TSB medium (BC-TSB) for the rapid enrichment of *B. cereus*, which significantly improved the pathogen’s detection efficiency—shortening the total detection cycle from the traditional 48 h to 6 h. It effectively inhibits non-target bacterial growth, accelerates *B. cereus* spore germination, and efficiently pre-enriches the pathogen in artificially contaminated dairy samples. In conclusion, BC-TSB has great potential for rapid *B. cereus* detection in food. Notably, the applicability of BC-TSB was only verified in dairy products, and its efficacy for ultra-low-concentration contamination needs optimization. Future research will focus on expanding its validation to more food matrices and improving sensitivity for low-concentration samples.

## Data Availability

The original contributions presented in the study are included in the article/Supplementary Materials; further inquiries can be directed to the corresponding authors.

## Supplementary Materials

The following supporting information can be downloaded at: https://www.mdpi.com/article/10.3390/foods15030466/s1, Table S1. Types and addition amounts of promoters and inhibitors. Table S2. Results of L16 (44) matrix orthogonal test.

## Abbreviations

The following abbreviations are used in this manuscript:

*B. cereus*	*Bacillus cereus*
TSB	Tryptic Soy Broth
PMAxx-qPCR	PMAxx-Quantitative Polymerase Chain Reaction
LB	Linear dichroism
BHI	Brain Heart Infusion
