# Supplementary material for: Establishing a Rapid Enrichment Medium for Bacillus cereus to Shorten Detection Time
Source: Foods. 2026 Jan 29;15(3):466. doi: 10.3390/foods15030466 (PMC12897033; doi:10.3390/foods15030466)
Supplement: Supplementary file 1 [file foods-15-00466-s001.zip › foods-4099680-supplementary.pdf]

Table S1. Types and addition amounts of promoters and inhibitors.

Factors	Added reagents	Concentration				
Promoters	Magnesium sulfate (g/L)	0.5	1.0	1.5	2.0	2.5
	Potassium dihydrogen phosphate (g/L)	1.0	2.0	3.0	4.0	5.0
	Ammonium sulfate (g/L)	5.0	10.0	15.0	20.0	25.0
	Manganese sulfate (g/L)	0.2	0.4	0.6	0.8	1.0
	Corn starch (g/L)	1.0	2.0	3.0	4.0	5.0
	Sodium dihydrogen phosphate (g/L)	1.0	3.0	5.0	7.0	9.0
	L-alanine (mmol/L)	20.0	40.0	60.0	80.0	100.0
	Inosine (mmol/L)	2.0	4.0	6.0	8.0	10.0
Inhibitors	Bile salt (g/L)	0.5	1.0	1.5	2.0	2.5
	Glycine (g/L)	5.0	7.0	9.0	11.0	13.0
	EDTA (g/L)	0.3	0.6	0.9	1.2	1.5
	Sodium deoxycholate (g/L)	0.5	1.0	1.5	2.0	2.5
	Nalidixic acid (mg/L)	1.0	3.0	5.0	7.0	9.0
	Sodium nitrite (mg/L)	100	200	300	400	500
	Magnesium chloride (g/L)	6.0	9.0	12.0	15.0	18.0
	Epsilon polylysine (g/L)	0.2	0.4	0.6	0.8	1.0
	Cinnamaldehyde (g/L)	0.12	0.24	0.36	0.48	0.60

Table S2. Results of L_{16} (4^4) matrix orthogonal test.

No.	A Inosine (mmol/L)		B Magnesium sulfate (g/L)	C Glycine (g/L)	D Sodium nitrite (g/L)	OD_{600}-vegetative cells	OD_{600}-spore
1	2.0		1.0	5.0	0.1	1.270	1.440
2	2.0		1.5	7.0	0.2	1.532	1.493
3	2.0		2.0	9.0	0.3	1.206	0.350
4	2.0		2.5	11.0	0.4	0.172	0.299
5	4.0		1.0	7.0	0.3	1.179	1.241
6	4.0		1.5	5.0	0.4	0.949	1.098
7	4.0		2.0	11.0	0.1	1.575	1.438
8	4.0		2.5	9.0	0.2	0.496	0.563
9	6.0		1.0	9.0	0.4	1.290	1.192
10	6.0		1.5	11.0	0.3	1.511	1.434
11	6.0		2.0	5.0	0.2	1.475	1.457
12	6.0		2.5	7.0	0.1	0.852	1.255
13	8.0		1.0	11.0	0.2	1.184	1.369
14	8.0		1.5	9.0	0.1	1.292	1.433
15	8.0		2.0	7.0	0.4	1.059	1.305
16	8.0		2.5	5.0	0.3	1.400	1.290
OD_{600}-vegetative cells	K1	4.18	4.92	5.09	4.99		
	K2	4.20	5.28	4.62	4.69		
	K3	5.13	5.32	4.28	5.30		
	K4	4.94	2.92	4.44	3.47		
extreme difference value		0.24	0.60	0.20	0.46		
Factors	B > D > A > C						
OD_{600}-spore	K1	3.58	5.24	5.29	5.57		
	K2	4.34	5.46	5.29	4.88		
	K3	5.34	4.55	3.54	4.32		
	K4	5.40	3.41	4.54	3.89		
extreme difference value		0.45	0.51	0.44	0.42		
Factors	B > A > C > D						
